# Innovating in CMHT's: mental health wellbeing group visits

**DOI:** 10.1192/bjo.2021.550

**Published:** 2021-06-18

**Authors:** Samuel Mammolotti Parkinson, Ismail Laher, Shola Johnson

**Affiliations:** Leeds & York Partnership NHS Foundation Trust

## Abstract

**Aims:**

‘Group consultations/visits’ are described as providing shared medical appointments delivering a range of care options and education by clinicians while providing elements of patient choice, empowerment and peer support.

This innovative and cost effective model of care delivery was first conceived in the US and has been gaining a strong foothold in the UK since 2016, mainly limited to GP settings.

The project goal was to attempt to transfer the model into a mental health setting by developing and delivering a novel intervention, to improve health and wellbeing options in a CMHT population.

**Method:**

A four session course was developed focussing on stress, sleep and nutrition. These chosen topics covered common significant challenges to patient health in psychiatry. Sessions were delivered to proactively address these important health related issues in a group visit setting.

Baseline and post intervention feedback including telephone interviews were conducted to evaluate the effectiveness of the intervention.

**Result:**

The qualitative data and the positive feedback obtained from participants indicate the intervention was highly valued and deemed effective in promoting positive health and lifestyle changes. Participants valued the educational and co-production aspects as well as the social and peer support elements of the groups. They appreciated the level of access they had with the clinicians involved, to explore their health and wellbeing in more detail without being limited by the usual 30 minute clinic follow-up sessions.

The clinicians involved found the sessions rewarding and more engaging than most of routine 1:1 clinic sessions as they were able to spend quality time exploring important issues and not just educate the patients but also be educated by their questions and feedback about their lived experiences.

**Conclusion:**

The project aim was met and we believe this intervention can be successfully incorporated into the identified service provision gap within the CMH T model. There is potential to build on and embed this innovation with roll-out to a wide range of service users in different settings.

In line with existing literature from GP settings, the consensus was that the amalgamated group visits/consultations model could be successfully modified to meet the needs of patients in the Mental Health arena who have a range of physical health and lifestyle concerns.

We planned to obtain more information about improvement in patient self-management but this was affected by the pandemic. However, we believe it is a cost effective and helpful innovation which warrants further promotion and evaluation.

